# From Plant Propagation to Anticancer Activity: Phytochemical and Biological Evaluation of Water Extracts of *Salvia aethiopis* L. Flowers, Leaves and Stems

**DOI:** 10.3390/molecules31101573

**Published:** 2026-05-08

**Authors:** Ani Georgieva, Inna Sulikovska, Maria Petrova, Vera Djeliova, Margarita Dimitrova, Lyudmila Dimitrova, Nenad Tsonevski, Teodor Badarov, Maria Lazarova, Petko Denev, Polina Petkova-Kirova, Krasimira Tasheva

**Affiliations:** 1 Department of Pathology, Institute of Experimental Morphology, Pathology and Anthropology with Museum, Bulgarian Academy of Sciences, Acad. G. Bonchev Str., 25, 1113 Sofia, Bulgaria; georgieva_any@abv.bg (A.G.); inna_sulikovska@ukr.net (I.S.); 2 Department of Plant Ecophysiology, Institute of Plant Physiology and Genetics, Bulgarian Academy of Sciences, Acad. G. Bonchev Str., 21, 1113 Sofia, Bulgaria; marry_petrova@yahoo.com (M.P.); mstoyadinova@abv.bg (M.D.); dim.lyudmila@gmail.com (L.D.); 3 Department of Molecular Biology of Cell Cycle, Institute of Molecular Biology “Acad. R. Tsanev”, Bulgarian Academy of Sciences, Acad. G. Bonchev Str, 21, 1113 Sofia, Bulgaria; vera@bio21.bas.bg; 4 Clinic of Thoracic Surgery, Military Medical Academy, G. Sofiyski Str., 3, 1606 Sofia, Bulgaria; nenadconevski@icloud.com (N.T.); tbadarov@abv.bg (T.B.); 5 Department of Synaptic Signaling and Communication, Institute of Neurobiology, Bulgarian Academy of Sciences, Acad. G. Bonchev Str., 23, 1113 Sofia, Bulgaria; m.lazarova@gmail.com; 6 Laboratory of Biologically Active Substances, Institute of Organic Chemistry with Centre of Phytochemistry, Bulgarian Academy of Sciences, 4000 Plovdiv, Bulgaria; petko.denev@orgchm.bas.bg

**Keywords:** *Salvia aethiopis*, in vitro plants, HPLC, cancer, fluorescence microscopy, flow cytometry

## Abstract

*Salvia aethiopis* L. is a medicinal and aromatic species of growing scientific interest due to its biological potential. The study aimed to develop an efficient in vitro micropropagation protocol and to evaluate the antioxidant and anticancer activity of aqueous extracts derived from the three different aerial plant parts—flowers, leaves and stems—of the in vitro-cultivated plants and compare them with those of the wild-growing plants. Optimal parameters for the micropropagation of the species were established, yielding 80% field survival and flowering in the second year. The highest total polyphenol and flavonoid content and antioxidant activity were recorded in the flower extract from wild plants—14,681 ± 211 mg GAE/100 g, 2317 ± 77 mg RE/100 g and 4563 ± 280 µmol TE/g, respectively. HPLC analysis confirmed the presence of key bioactive compounds, including rosmarinic acid, caffeic acid, and apigenin. The anticancer potential of the different extracts was assessed against human cervical, mammary and colorectal cell lines. The extracts exhibited significant antiproliferative activity, with HT-29 colorectal carcinoma cells being the most sensitive. Flower extracts of wild plants showed the strongest cytotoxic effects with IC_50_ values at 72 h being lower than 100 μg/mL for all cancer cell lines. Fluorescence microscopy and flow cytometry analyses indicated that the observed extract-induced anticancer effects were associated with mitochondrial dysfunction, cell cycle alterations, modulation of autophagy, and induction of apoptotic and necrotic cancer cell death. These findings highlight the potential of extracts of *S. aethiopis* for anticancer therapy.

## 1. Introduction

Cancer remains one of the leading causes of morbidity and mortality worldwide, with incidence rates continuously rising due to population aging, environmental exposure, and lifestyle-related risk factors [1]. The disease is characterized by uncontrolled cell proliferation, resistance to apoptosis, metastatic potential and sustained angiogenesis of the tumors [2]. Despite advances in conventional therapies, limitations such as systemic toxicity, drug resistance, and limited selectivity highlight the need for safer and more effective therapeutic agents without side effects [3].

Plants are widely used as food additives, spices, and therapeutic agents, largely owing to their rich phytochemical composition, including phenolic acids, flavonoids, terpenoids, and essential oils [4]. Medicinal plants play a central role in anticancer drug discovery as well, providing structurally diverse bioactive molecules with multitarget mechanisms of action [5]. Plant in vitro culture systems are gaining increasing recognition as they offer key advantages such as rapid, large-scale propagation of disease- and pathogen-free plants of constant composition and enhanced biosynthesis of therapeutically active secondary metabolites [6,7,8]. Thus, extracts from in vitro-obtained plants have increasingly been evaluated for their cytotoxic and antiproliferative effects against various types of tumors [9].

The Lamiaceae family represents an important group of aromatic and medicinal plants known for their high content of bioactive metabolites and diverse pharmacological properties [10,11]. The genus Salvia, one of the largest genera within Lamiaceae, comprises nearly 1000 species distributed worldwide and is traditionally used for the treatment of inflammatory disorders, infections, and conditions associated with oxidative stress [12,13]. Increasing evidence indicates that Salvia species, in addition to their antioxidant and anti-inflammatory properties, exhibit antiproliferative, and proapoptotic effects in malignant cells as well, suggesting their potential relevance in cancer-related research [14].

*Salvia aethiopis* L., commonly known as Ethiopian or Mediterranean sage, is a perennial species whose anticancer activity remains insufficiently explored and is mostly limited to organic solvent extracts of the plant [15,16]. Published studies, however, report an abundance of water soluble polyphenolic acids such as rosmarinic, caffeic, ferulic, p-coumaric and malic acids as well as salvianic acid A (danshensu) and C and salvianolic acid K [15,17,18,19], compounds widely associated with antioxidant activity and modulation of cancer-related cellular processes [20].

The aim of the present study was to analyze the anticancer properties of water extracts of in vitro-cultivated plants, separately for the three distinct aerial organs—flowers, leaves, and stems—against three cancer cell lines (HeLa, HT 29, and MCF 7) and to compare them with the anticancer properties of the respective aerial parts of wild growing plants. The total polyphenol and flavonoid content were also determined, as well as the content of the main polyphenolic acids such as rosmarinic and caffeic acid as well as of the flavonoid apigenin. Antioxidant activity (ORAC and HORAC) was also assessed.

## 2. Results

### 2.1. In Vitro Plant Culture

The collected seeds were surface sterilized using three procedures. Effective seed sterilization (100%) was achieved by successively immersing seeds in 70% ethanol for 2 min and 20% bleach ACE^®^ containing <5% active chlorine for 10 min, followed by three rinses with distilled water. Using a combination of 70% ethanol and 0.1% HgCl_2_, 100% seed sterilization was also achieved. The sterilization with 100% bleach solution was also successful but failed to produce any viable seedlings and is not appropriate for *S. aethiopis* seed sterilization.

In the present experiments using Murashige and Skoog [21] medium containing vitamins, without the addition of plant growth regulators and fortified with 0.6% agar, 100% seed germination was detected.

#### 2.1.1. Shoot Induction and Multiplication

Shoot induction was observed on all tested media supplemented with plant growth regulators (PGRs) within four weeks of culture. In contrast, the control Murashige and Skoog medium [21] lacking PGRs did not induce shoot proliferation. The effects of individual cytokinins, kinetin (K) and 6-benzylaminopurine (BAP), on shoot induction were evaluated. Kinetin demonstrated higher morphogenic efficiency compared to BAP. Explants cultured on MS medium supplemented with 1 mg/L K exhibited 70% shoot induction, with a mean of 2.64 shoots per explant and an average shoot height of 2.80 cm. A comparable shoot induction frequency (70%) was recorded on medium containing 1 mg/L BAP; however, the mean number of shoots per explant was significantly lower (1.71 ± 0.16).

A synergistic effect between cytokinins and auxins on shoot multiplication from nodal explants of *S. aethiopis* was observed. Combinations of cytokinins with indole-3-acetic acid (IAA) produced better results than combinations with indole-3-butyric acid (IBA). The most effective PGR treatment consisted of 1 mg/L K combined with 0.1 mg/L IAA, resulting in an average of 4.00 ± 0.17 shoots per nodal explant and a mean shoot height of 3.60 cm.

**Figure 1:**
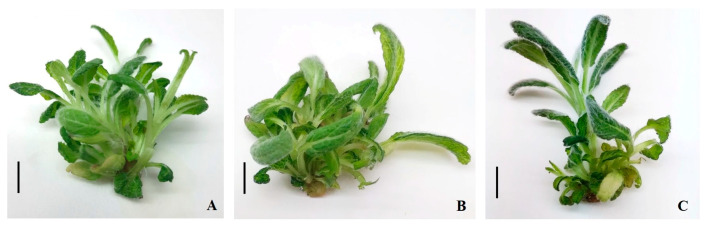
Effect of different nutrient media on *S. aethiopis* shoot multiplication. (**A**) K_1_ + IAA_0.1_; (**B**) BAP_1_ + IAA_0.1_; (**C**) Z_1_ + IAA_0.1_. Scale bar (**A**–**C**) = 1.0 cm.

Media supplemented with BAP and IAA, as well as zeatin (Z) and IAA, also enhanced shoot multiplication, yielding between 3.33 ± 0.11 and 3.61 ± 0.18 shoots per explant. Shoots regenerated on kinetin- and zeatin-containing media were vigorous and exhibited normal green morphology, whereas some shoots cultured on BAP-containing media showed hyperhydricity. In contrast, media containing combinations of BAP, zeatin, or kinetin with IBA were less effective for shoot multiplication (Table 1, Figure 1).

#### 2.1.2. In Vitro Rooting and Acclimation

**Figure 2:**
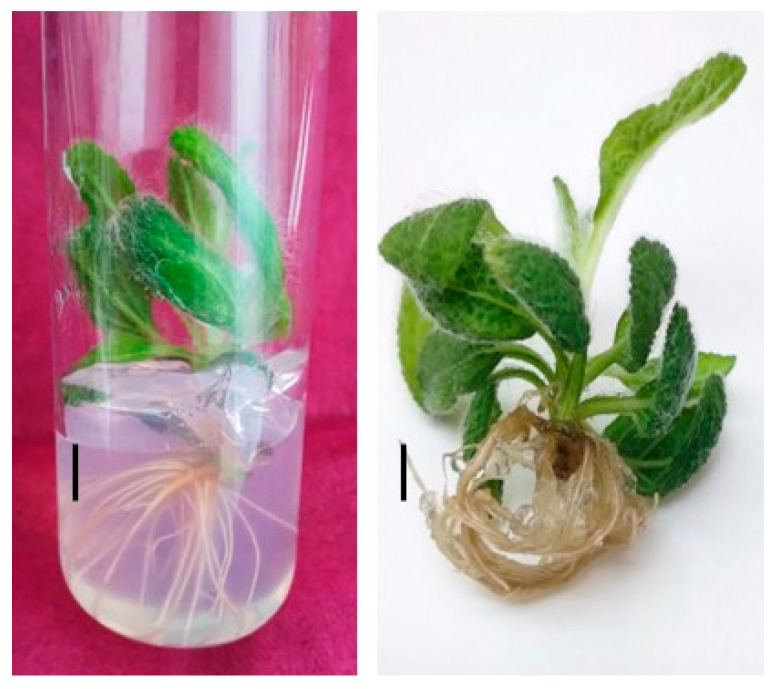
In vitro rooting of *S. aethiopis* on ½ MS medium, containing 0.25 mg/L IBA. Scale bar = 0.5 cm.

Shoots were separated from one another and placed on half strength MS media with varying concentrations of IBA to promote rooting. Additionally, a nutrient medium that included both IAA and IBA was tested. The rooting of the obtained shoots proved to be one of the most crucial phases in the in vitro culture of Salvia plants. Obtaining a good root system is also particularly important for the subsequent adaptation of the plants to external conditions. The beginning of root formation was observed 15 days after the shoots were planted. Mass rooting was recorded after 30–35 days. In all nutrient media we recorded an increase in the height of the plants by 1–2 cm. The efficiency of rhizogenesis varied between 20% and 50% in the culture media studied. MS half strength medium fortified with 0.2 mg/L IBA resulted in the maximum number of roots (4.50 ± 0.16) with the average length of the induced roots being 3.6 ± 0.17 cm after a culture period of 4 weeks. Lower rooting response was reported at higher and lower concentrations of IBA. The second in effectiveness is a nutrient medium containing 0.2 mg/L IAA and 2 mg/L IBA which induced 40% root formation with 2.37 roots per explant (Table 2, Figure 2).

Successful ex vitro acclimation requires in vitro-obtained plants to possess a well-developed root system and gradual and carefully controlled reduction in humidity. The composition of the potting substrate also plays a critical role in plant adaptation during the acclimation phase. In the present study, the optimal hardening substrate consisted of soil, sand, and perlite in a ratio of 1:1:1 (*v*/*v*/*v*), providing adequate drainage conditions and resulting in a high survival rate (80%) of micropropagated plants.

**Figure 3:**
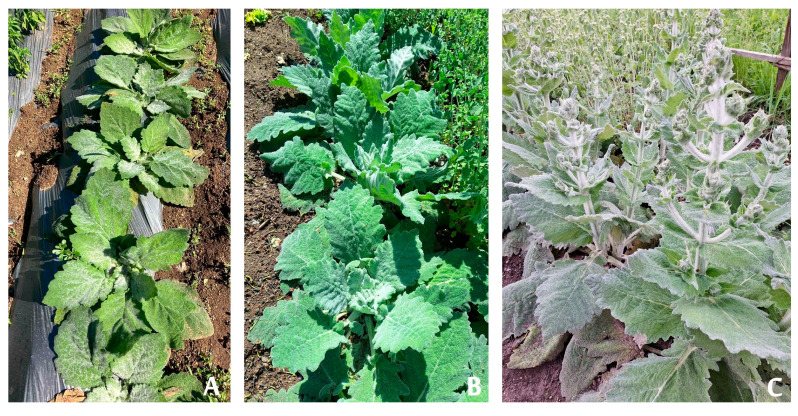
Ex vitro adapted plants in experimental field: (**A**,**B**) during first year of cultivation; (**C**) bloomed plants in the second year.

Following successful transfer to field conditions, the plants continued normal growth and development (Figure 3). The acclimatized plants were vigorous and formed numerous large, gray-green leaves. Figure 3A,B show the plants during the first year of field cultivation. Flowering was initiated in June of the second year after transplantation.

### 2.2. Phytochemical Analysis

#### 2.2.1. Content of Total Polyphenols, Total Flavonoids and Antioxidant Activity

The obtained results demonstrate clear differences in the content of phenolic compounds and antioxidant capacity depending on both plant organ and plant origin (in vitro-derived field-grown plants vs. wild plants). The highest total polyphenol content was recorded in flower extract from wild plants—14,681 ± 211 mg GAE/100 g DW—followed by the stem extract from wild plants—11,756 ± 255 mg GAE/100 g DW—and leaf extract from in vitro-derived field-grown plants—11,127 ± 148 mg GAE/100 g DW. In general, the flower extract contained higher levels of total polyphenols compared with leaves and stems, particularly in wild plants. A similar trend was observed for the total flavonoid content. Flowers from wild plants exhibited the highest flavonoid content—2317 ± 77 mg RE/100 g DW, whereas the stems from in vitro-derived plants showed markedly lower levels 178 ± 17 mg RE/100 g DW. The leaves from both types of plants contained intermediate amounts of flavonoids, with higher values in in vitro-derived leaves. These findings indicate that flowers are the richest source of flavonoids among the examined organs. The antioxidant capacity, assessed by ORAC and HORAC assays, corresponded well with the phenolic profiles. The highest ORAC value was measured in flower extract from wild plants—4563 ± 280 µmol TE/g DW) while the lowest was recorded in stems from in vitro-derived plants—3001 ± 111 µmol TE/g DW (Table 3). Overall, the results indicate that the plant organ significantly influences the accumulation of phenolic compounds and antioxidant capacity, with flower extracts showing the highest levels, followed by leaves and stems. Moreover, wild plants tend to exhibit enhanced phenolic content and antioxidant activity compared to in vitro-derived field-grown plants. The positive association between total polyphenols, total flavonoids, and antioxidant capacity suggests that phenolic compounds are major contributors to the observed radical scavenging and hydroxyl radical averting activities.

#### 2.2.2. HPLC Analysis

The highest concentration of caffeic acid was identified in the flower samples of wild species, followed by the leaves of wild species and the flowers of cultivated plants. Regarding the content of rosmarinic acid, the highest levels were detected in the leaves of wild species (5157.1 ± 255.9). Apigenin was found in the greatest amount in the leaf samples of wild species (268.8 ± 18.5), followed by the leaves of cultivated plants (75.4 ± 7.0) and the flowers of wild species (51.1 ± 2.6) (Table 4).

### 2.3. Antitumor Activity

#### 2.3.1. Cell Viability Assay

**Figure 4:**
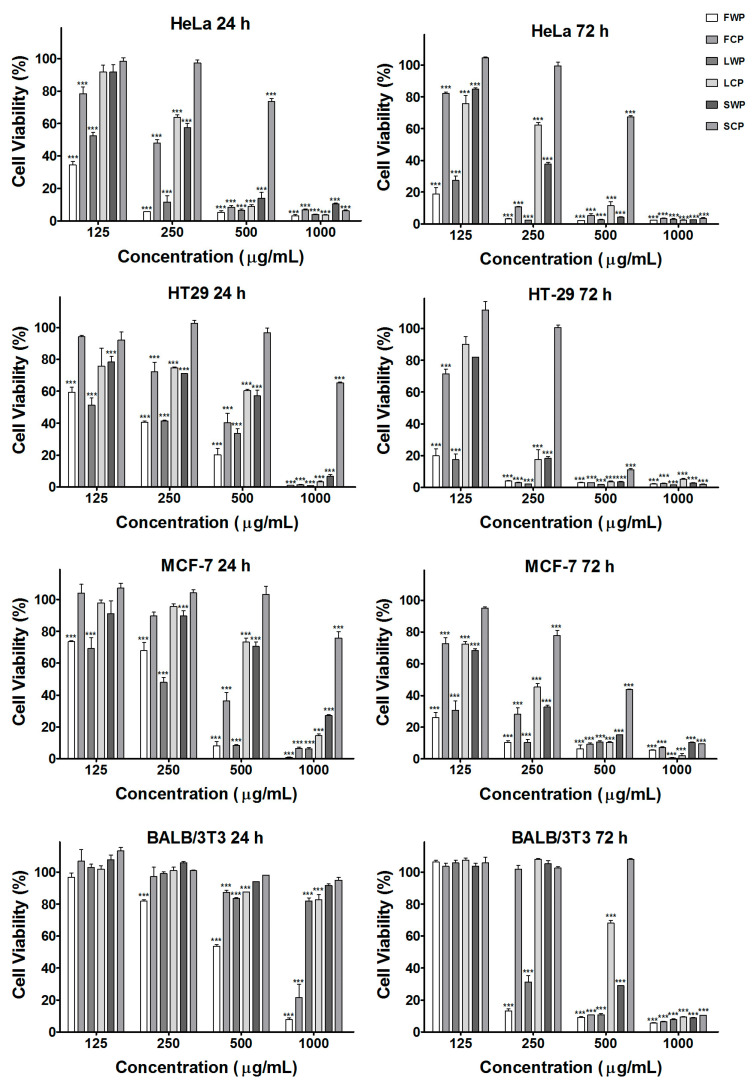
Effects of extracts derived from flowers, leaves, and stems of in vitro-cultured and wild-growing *S. aethiopis* plants on the viability of HeLa, HT-29, and MCF-7 and BALB/3T3 cells determined by the MTT assay after 24 and 72 h. FC (flowers of in vitro-cultivated plants); FW (flowers of wild plants); LC (leaves of in vitro-cultivated plants); LW (leaves of wild plants); SC (stems of in vitro-cultivated plants); SW (stems of wild plants). Data are presented as mean ± SD; *** *p* < 0.001.

The antiproliferative activity of extracts derived from both in vitro-propagated and wild-growing plants was examined in the human cancer cell lines HeLa, HT-29, and MCF-7 as well as in the control noncancerous cell line BALB/3T3 using the MTT viability assay following 24 and 72 h of exposure (Figure 4).

In untreated control cells, high metabolic activity was observed, corresponding to normal cell viability and proliferation. In contrast, treatment with the tested plant extracts resulted in a concentration-dependent decrease in cell viability in all examined cancer cell lines. The inhibitory effect became more pronounced after 72 h of exposure compared with 24 h.

The mean IC_50_ values of the tested *S. aethiopis* extracts determined by nonlinear regression curve fit analyses of the cell viability data are presented in Table 5.

Among the tested samples, extracts obtained from flowers exhibited the strongest antiproliferative activity, particularly those derived from wild-growing plants. In HeLa cells, the IC_50_ values decreased from 98.7 μg/mL at 24 h to 73.8 μg/mL at 72 h for wild plant extracts, whereas extracts from in vitro-propagated plants showed lower potency (228.2 μg/mL at 24 h and 167.5 μg/mL at 72 h). A similar trend was observed in HT-29 cells, where the wild plant flower extract demonstrated markedly higher activity, with IC_50_ values decreasing from 175.2 μg/mL at 24 h to 50.4 μg/mL at 72 h.

In MCF-7 breast cancer cells, a moderate antiproliferative effect was detected, again with stronger inhibition observed after 72 h exposure. Extracts obtained from leaves and stems generally exhibited weaker cytotoxic activity compared with flower extracts, particularly those derived from in vitro-propagated plants.

In contrast, the tested extracts showed substantially lower cytotoxicity toward the noncancerous BALB/3T3 fibroblast cell line. The IC_50_ values for these cells were considerably higher than those observed for tumor cells, indicating a degree of selectivity toward cancer cells.

These results demonstrate that *S. aethiopis* extracts exert significant antiproliferative effects in human cancer cell lines in a time-dependent manner, with flower extracts from wild-growing plants displaying the highest cytotoxic potency.

#### 2.3.2. Fluorescent Microscopy Cytomorphological Analysis of *S. aethiopis*-Treated HT-29 Cancer Cells

**Figure 5:**
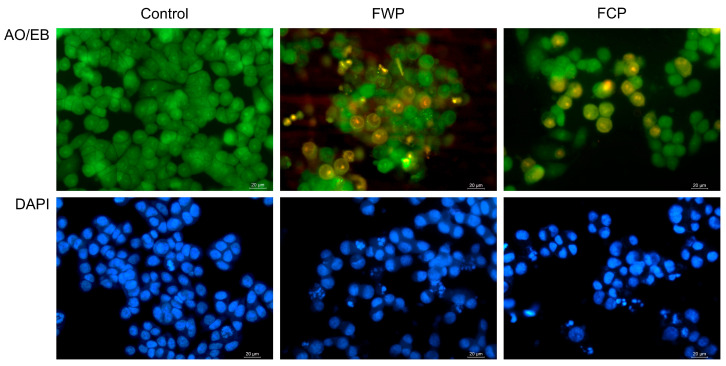
Fluorescence microscopy images of the morphological and nuclear changes induced by flower extracts of wild-growing and in vitro-propagated *S. aethiopis* plants in HT-29 colorectal carcinoma cells. Control—untreated cells; FWP—cells treated with extract from flowers of wild plants; FCP—cells treated with extract from flowers of in vitro-cultivated plants; AO/EB—acridine orange/ethidium bromide staining; DAPI—staining with 4′,6-diamidino-2-phenylindole. Images were acquired by fluorescence microscopy at 40× magnification.

Morphological and nuclear changes induced by flower extracts obtained from wild-growing and in vitro-propagated *S. aethiopis* plants in HT-29 colorectal carcinoma cells were evaluated using fluorescence microscopy following acridine orange/ethidium bromide (AO/EB) and DAPI staining (Figure 5).

In the untreated control, cells exhibited normal morphology with intact plasma membranes and uniformly stained nuclei, indicating viable cells with no signs of nuclear condensation or fragmentation.

Following treatment with extracts obtained from wild-growing plants (FWP), marked morphological alterations were observed. AO/EB staining revealed an increased number of cells exhibiting orange fluorescence, indicating compromised membrane integrity and apoptotic cell death. In addition, nuclear condensation and chromatin fragmentation were evident in DAPI-stained cells, suggesting the induction of apoptosis.

Similarly, treatment with extracts derived from in vitro-propagated plants (FCP) resulted in pronounced cytomorphological changes compared with untreated controls. Cells displayed increased orange fluorescence and chromatin condensation. DAPI staining revealed fragmented and condensed nuclei, characteristic of apoptotic cells.

#### 2.3.3. Annexin V/PI Flow Cytometric Analysis

**Figure 6:**
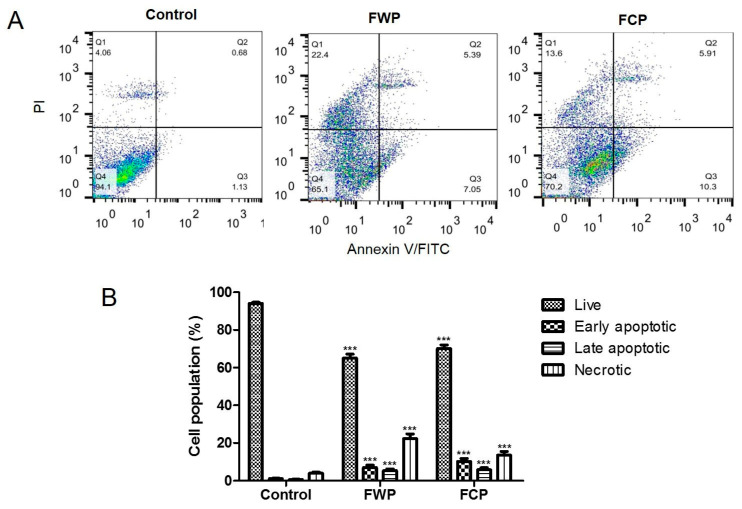
Effect of flower extracts of wild-growing and in vitro-propagated *S. aethiopis* plants on the apoptosis/necrosis in HT-29 colorectal carcinoma cells assessed by Annexin V/PI flow cytometric analysis. (**A**) Representative dot plots of control untreated cells and extract-treated cells. (**B**) Quantitative analysis of cell populations expressed as percentage of total cells. Data are presented as mean ± SD from three independent experiments. Statistics: one-way ANOVA, followed by Bonferroni test. *** *p* < 0.001 compared to the untreated control.

The induction of apoptosis in HT-29 colorectal carcinoma cells following treatment with flower extracts obtained from wild-growing and in vitro-propagated *S. aethiopis* plants was evaluated by Annexin V/propidium iodide (PI) double staining and flow cytometry (Figure 6A).

Flow cytometric analysis using Annexin V/PI staining revealed a marked increase in apoptotic and necrotic cell populations in HT-29 cells following treatment with flower extracts from *S. aethiopis* compared with untreated controls. In control cells, the vast majority (94.1%) of the population remained viable, with only minimal proportions of early apoptotic, late apoptotic, and necrotic cells (1.13%, 0.68% and 4.6%, respectively), indicating low basal levels of cell death (Figure 6A). Treatment with extract from wild-growing plants (FWP) resulted in a pronounced reduction in viable cells (65.1%), accompanied by a substantial increase in necrotic cells (22.4%). In addition, both early and late apoptotic populations were elevated compared with the control (7.05% and 5.39%, respectively), indicating induction of cell death involving both apoptotic and necrotic components. Similarly, treatment with extract from in vitro-propagated plants (FCP) decreased cell viability (70.2%) and led to an increase in early apoptotic cells and late apoptotic cells (10.3% and 5.91%, respectively). Notably, the proportion of necrotic cells was lower than that observed in FWP-treated cells (13.6%) but remained significantly higher than in control cells (Figure 6A). Statistical analysis confirmed significant differences in the proportions of viable, apoptotic, and necrotic cell populations in both wild and in vitro-cultivated plants (*p* < 0.001 compared to untreated control; Figure 6B). These results demonstrate that the observed reduction in cancer cell viability after treatment with flower extracts of *S. aethiopis* is associated with apoptotic and necrotic cell death.

#### 2.3.4. Cell Cycle Analysis

**Figure 7:**
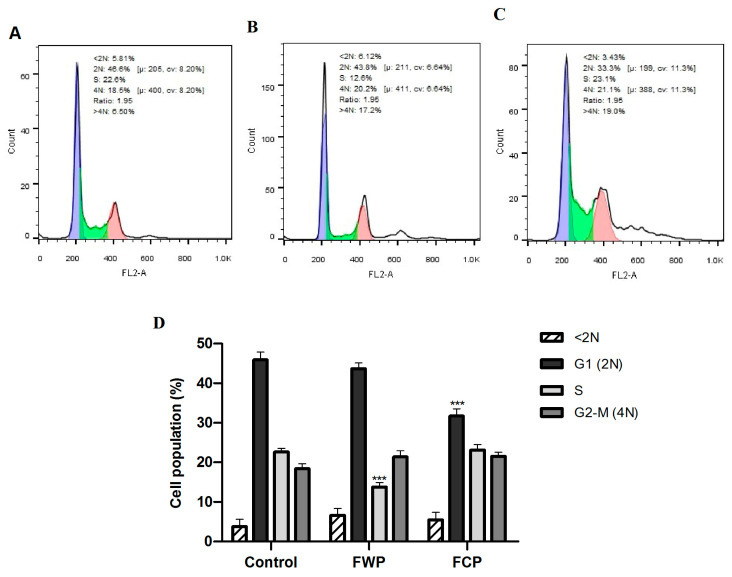
Effects of flower extracts obtained from wild-growing and in vitro-propagated *S. aethiopis* plants on cell cycle progression of HT-29 colorectal carcinoma cells. (**A**) Untreated cells; (**B**) cells treated with extracts from wild-growing plants; (**C**) cells treated with extracts from in vitro-propagated plants; (**D**) bar chart illustrating the distribution of cells across different cell cycle phases. Values are expressed as mean ± SD from three independent experiments; *** *p* < 0.001 versus control.

The effects of flower extracts obtained from wild-growing and in vitro-propagated *S. aethiopis* plants on cell cycle progression in HT-29 colorectal carcinoma cells were evaluated by flow cytometry (Figure 7A–D).

In untreated control cells (Figure 7A), the majority of the cell population was distributed in the G1 phase (48.6%), followed by the S phase (22.6%) and the G2/M phase (18.5%), with a small proportion of cells detected in the sub-G1 fraction (5.81%), indicating low basal apoptosis.

Treatment with extracts from wild-growing plants (Figure 7B) significantly altered cell cycle distribution. A marked reduction in the S-phase population was observed (12.6% vs. 22.6% in control; *** *p* < 0.001), accompanied by an increase in the proportion of G2/M cells (20.2%) and a moderate decrease in the G1 fraction (43.8%). Additionally, a significant increase in the >4 N population (17.0%) compared to the control (6.5%) was also detected, suggesting the occurrence of replication stress, mitotic slippage or defective mitotic progression.

In contrast, extracts derived from in vitro-propagated plants (Figure 7C) produced a distinct pattern of cell cycle modulation. A pronounced reduction in the G1 population was observed (33.3% vs. 48.6% in control; *** *p* < 0.001), coupled with a moderate increase in the G2/M population (21.1%). Notably, the increase in the >4 N population (19.0%) was even more pronounced than that observed in cell cultures treated with the wild plant extract.

Quantitative analysis of three independent experiments confirmed that wild-growing plant extracts significantly reduced the S-phase cell population (*** *p* < 0.001), whereas in vitro-propagated plant extracts significantly decreased the G1 fraction (*** *p* < 0.001) compared with untreated controls (Figure 7D).

These results demonstrate that flower extracts from *S. aethiopis* differentially modulate cell cycle progression in HT-29 cells depending on the plant source, with wild-growing plant extracts primarily reducing S-phase cells and in vitro-propagated plant extracts mainly decreasing G1-phase cells and inducing higher polyploidy, suggesting replication stress, checkpoint dysregulation, and perturbation of mitotic progression.

#### 2.3.5. Mitochondrial Membrane Potential

**Figure 8:**
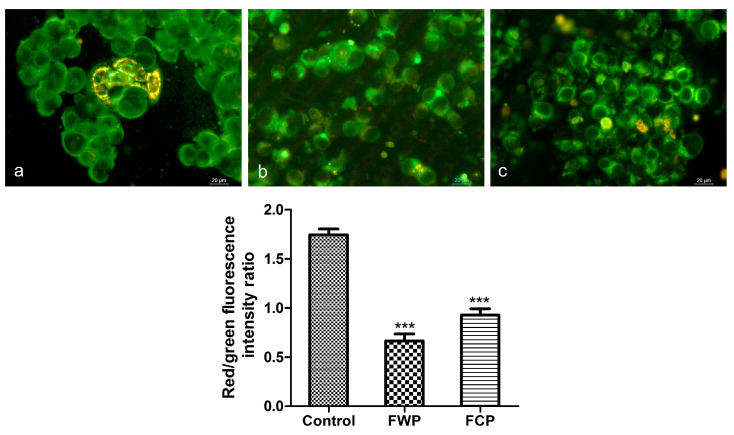
Changes in mitochondrial membrane potential (ΔΨm) in HT-29 cells following 24 h treatment with extracts of wild-growing and in vitro-cultivated *S. aethiopis* plants assessed by JC-1 staining. (**a**)—control untreated cells; (**b**)—cells treated with wild plant extract (FWP); (**c**)—cells treated with extract of in vitro-cultivated plants (FCP). Data are presented as mean ± SD; *** *p* < 0.001.

Changes in mitochondrial membrane potential (ΔΨm) were evaluated in HT-29 cells using JC-1 staining after 24 h extract treatment (Figure 8). Fluorescence images were captured by fluorescence microscopy, and mitochondrial depolarization was assessed by calculating the ratio of red (JC-1 aggregates) to green (JC-1 monomers) fluorescence intensity.

JC-1 staining revealed heterogeneous mitochondrial polarization in control cells, with clusters of cells displaying strong red mitochondrial fluorescence (Figure 8a). Following treatment with the plant extracts, a visible reduction in red fluorescence intensity and in the number of cells exhibiting red mitochondrial aggregates was observed, indicating mitochondrial depolarization (Figure 8b,c). The wild plant extract exerted a more pronounced effect on mitochondrial membrane potential compared to the extract from in vitro-cultivated plants, as indicated by a greater reduction in the red/green fluorescence ratio relative to the control. Quantitative analysis of the fluorescence intensity data indicated statistically significant decrease in the red/green ratio in cells treated with both extracts compared to untreated controls (*** *p* < 0.001).

#### 2.3.6. Assessment of Autophagy-Related Vesicular Staining

**Figure 9:**
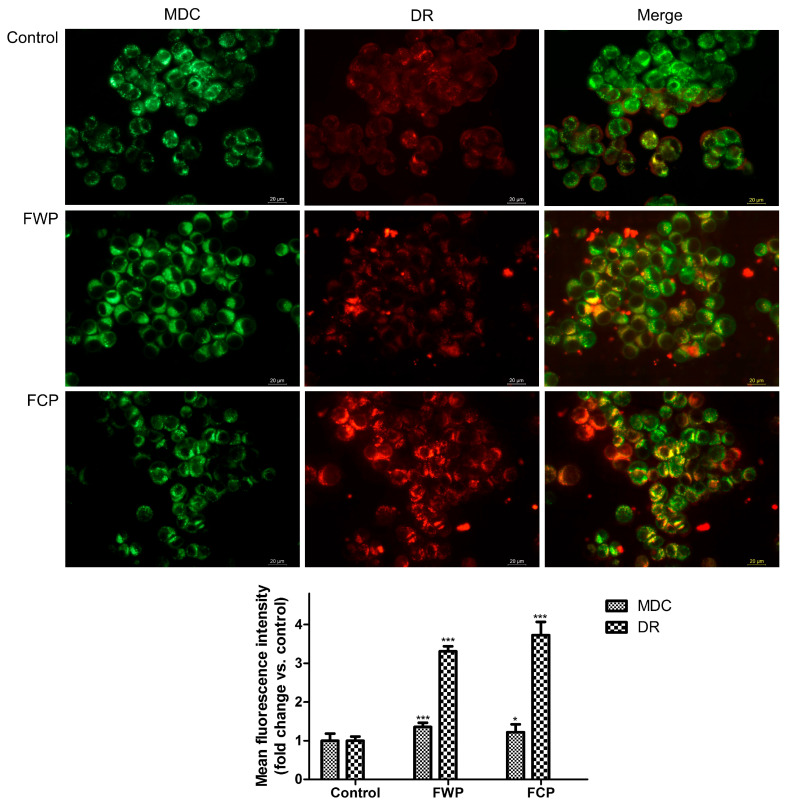
Induction of autophagy in HT-29 colorectal carcinoma cells following treatment with flower extracts from wild-growing and in vitro-propagated *S. aethiopis* plants. Autophagic vacuoles were visualized by MDC staining (green), while acidic lysosomal compartments were detected using LysoTracker Deep Red (DR, red). Images were acquired by fluorescence microscopy; scale bar = 20 µm. Quantitative analysis data are presented as mean ± SD; *** *p* < 0.001; * *p* < 0.05.

Autophagy induction in HT-29 cells following treatment with flower extracts from wild-growing and in vitro-propagated *S. aethiopis* plants was evaluated by fluorescence microscopy using monodansylcadaverine (MDC) and LysoTracker Deep Red (DR) staining (Figure 9). MDC preferentially accumulates in autophagic vacuoles due to its affinity for autophagic membranes, while DR labels acidic compartments such as lysosomes and autolysosomes. The simultaneous visualization of MDC-positive vesicles and DR fluorescence allows detection of autophagic structures and lysosomal activity associated with autophagy.

Fluorescence microscopy analysis of HT-29 cells stained with MDC and DR revealed treatment-related changes in vesicular fluorescence patterns. DR fluorescence intensity increased significantly compared with control cells, reaching 3.3-fold (*p* < 0.001) and 3.7-fold (*p* < 0.001) elevation in cells treated with FWP and FCP extracts, respectively. In contrast, MDC staining displayed only moderate differences between the control and treated cells. The green fluorescence intensity observed following exposure to *S. aethiopis* extracts showed an increase of 1.4-fold (*p* < 0.001) for FWP and 1.2-fold (*p* < 0.05) for FCP, as compared to the control. Merged images revealed an increased overlap between MDC and DR fluorescence signals in treated cells compared with control cells, indicating partial colocalization of MDC-positive vesicles with acidic lysosomal compartments. The strong increase in DR fluorescence together with the modest elevation of MDC staining and the enhanced MDC/DR overlap suggests that treatment with *S. aethiopis* extracts may promote the formation or maturation of acidic vesicular compartments, a process that can be associated with autophagy-related vesicular trafficking. Nevertheless, additional molecular markers would be required to confirm the extent of autophagy induction.

## 3. Discussion

In this study, an effective system for the in vitro propagation of *S. aethiopis* was developed, demonstrating high morphogenetic potential while clearly revealing species-specific responses to plant growth regulators (PGRs). Although the general sequence of regeneration processes corresponds to established patterns of micropropagation in the genus Salvia, certain physiological characteristics—particularly the preference for specific cytokinins and rooting efficiency—indicate that regeneration in *S. aethiopis* is regulated by a specific hormonal balance requiring species-specific optimization.

The successful establishment of aseptic cultures is a key prerequisite for reproducible in vitro regeneration. The complete seed sterilization achieved through sequential ethanol and sodium hypochlorite treatment confirms the effectiveness of combined sterilization strategies widely applied in plant tissue cultures, including Salvia species, where ethanol pre-treatment enhances disinfectant penetration and sodium hypochlorite ensures efficient microbial elimination [22,23].

Achieving 100% germination on MS medium without added hormones indicates effective endogenous regulation in the early stages of plant development. Similar results have been reported for other members of the genus Salvia, in which seeds successfully germinate in vitro on basal MS medium without exogenous growth regulators [24]. This supports the view that the initial phase of germination within the genus is largely independent of externally applied phytohormones. However, the lack of analysis of endogenous hormones limits the interpretation of whether the observed effect is due to intraspecific physiological regulation or to a favorable physiological state of the seeds.

In our study, bud induction of *Salvia aethiopis* was observed only in media containing cytokinins, confirming their central role in the activation of axillary meristems and the acquisition of organogenic competence [25,26]. A similar dependence on cytokinins has been widely described in various Salvia species, in which bud formation does not occur in the absence of these regulators [23,27,28,29]. In the present study, kinetin demonstrated higher morphogenetic efficacy compared to BAP, leading to both a greater number of new shoots and better growth in height. This result contrasts with the prevailing literature on the micropropagation of Salvia species, in which BAP is generally reported as the most effective cytokinin for multiplication [23,28,29,30,31,32,33]. The increased responsiveness to kinetin likely reflects species-specific differences in the sensitivity of cytokinin receptors in *S. aethiopis*, as well as in the uptake or metabolism of the hormone by the plant [34]. This effect may also be considered advantageous, as cytokinins—particularly in Salvia species—not only promote shoot proliferation but have been shown to influence the balance of organogenic differentiation and physiological stability in vitro, as demonstrated in *Salvia bulleyana* tissue cultures [34]. This effect also highlights the limitations of directly transferring in vitro culture protocols between closely related species. In this case, BAP concentrations effective in other Salvia species may exceed the optimal hormonal threshold for *S. aethiopis* and lead to hyperhydration, as documented with the use of high BAP concentrations in tissue cultures [35,36].

The increase in the multiplication coefficient following the addition of low concentrations of auxins, IAA and IBA confirms the classic auxin–cytokinin interaction that stimulates organogenesis [25]. The most effective combination in *S. aethiopis*—kinetin with IAA—demonstrates a clear synergistic effect between the two plant growth regulators, also observed in other Salvia species [27,31]. The better response to IAA compared to IBA is particularly interesting. While IBA often stimulates morphogenesis in various medicinal plants [25], its lower efficacy in bud proliferation in *S. aethiopis* suggests species-specific differences in auxin metabolism and signaling. IAA is rapidly metabolized, whereas IBA functions as a more stable precursor [37]. Consequently, the results indicate that dynamic auxin homeostasis is more favorable for organogenesis in *S. aethiopis*.

Despite the efficient proliferation of shoots, rooting in *S. aethiopis* remains relatively limited, confirming that rhizogenesis is a critical stage in the micropropagation of the genus Salvia [38,39,40]. A 1/2 MS medium with low IBA concentration is also optimal for rooting in other Salvia species [31,33,39,41], indicating that the results for *S. aethiopis* follow a physiological pattern common to the genus. However, the still moderate rooting rate suggests that auxin concentration is not the sole determining factor influencing rooting. Carbohydrate supply, endogenous auxin transport, and accumulated physiological stress during multiplication likely play a role [38,39,40,42].

The high survival rate during acclimatization indicates the good physiological condition of regenerated plants. The soil-sand-perlite substrate (1:1:1) provides optimal aeration and drainage—clearly key factors for the successful adaptation of *S. aethiopis*. Normal growth and the onset of flowering in the second year indicate the functional stability of the plants.

In summary, *S. aethiopis* exhibits high regenerative potential under in vitro conditions but demonstrates specific hormonal requirements compared to other studied Salvia species. The higher efficacy of kinetin compared to BAP calls into question the traditionally accepted cytokinin hierarchy within the genus and highlights the need for species-specific optimization of protocols. The results obtained contribute to a deeper understanding of hormonal regulation in the micropropagation of Salvia and lay the foundation for future biotechnological applications related to the conservation and sustainable cultivation of the species and the production of bioactive compounds.

Existing research on the anticancer properties of *S. aethiopis* L. is limited and focuses primarily on extracts produced using organic solvents [15,16]. Moreover, until recently, no investigations had assessed the anticancer potential of *S. aethiopis* cultivated under in vitro conditions. In our recent study, we examined the effects of aqueous extracts derived from whole in vitro-grown *S. aethiopis* plants on three human cancer cell lines—lung carcinoma A549, prostate adenocarcinoma PC 3, and hepatocellular carcinoma HepG2 [43].

In the present study we extended and deepened our research by separately considering the aqueous extracts of all three aerial parts (flowers, leaves and stems) of both wild and in vitro-grown plants. We evaluated and compared the phytochemical composition of all six extracts by examining the total polyphenol and flavonoid content and by performing HPLC analysis of the content of the main phenolic compounds such as rosmarinic acid, caffeic acid and apigenin; the antioxidant activity of all the extracts was also studied. Moreover, across all six extracts we assessed and compared the anticancer potential of the extracts against three additional cancer cell lines (HeLa, HT 29, and MCF 7) and further analyzed the mechanisms behind the anticancer effects by examining extract-induced apoptotic events and cell cycle alterations and additionally assessing changes in the mitochondrial membrane potential (ΔΨm) and evaluating authophagy-related processes for both wild-growing and in vitro-cultivated plants.

All six tested extracts demonstrated notable antiproliferative effects against all three cancer cell lines with differences depending on the plant organ (leaf, flower, stem), cultivation type (wild-growing vs. in vitro-cultivated), exposure time, and cell line sensitivity. Most efficient was the extract from flowers of wild plants, followed closely by the extract of leaves from wild plants. Such a result is not surprising as the extracts from flowers of wild plants show the highest total polyphenols and total flavonoids content, as well as antioxidant ORAC and HORAC activity compared to all other extracts. Consistently second in place for those parameters are the leaves of wild plants, slightly exceeding the respective parameters in the flowers of in vitro-grown plants. Polyphenols, including flavonoids, are well known to exert preventive and therapeutic anticancer effects through several interconnected biological mechanisms that span antioxidant defense, inflammation control, modulation of cancer-related signaling pathways and direct effects on tumor cell fate [44,45,46,47]. As for the antioxidant activity, although the relationship between antioxidant activity and anticancer effect is certainly not absolute [48], oxidative stress has been increasingly recognized as a key factor in cancer initiation and progression [49,50]. Antioxidants, in addition to directly neutralizing free radicals and reducing oxidative stress, have been shown to target different signaling pathways to attenuate proliferation, survival, and invasion of malignant cells and endorse cell death pathways to destroy cancer cells [51,52].

Analyzing the differences in the effects of the extracts across cancer cell lines, the colorectal cancer cell line (HT-29) was the most sensitive to the extracts, followed by the breast (MCF-7) and cervical (HeLa) carcinoma cell lines, with IC_50_ values of 50.4, 65.7, and 73.8 μg/mL for the FWP extract and 67.01, 74.9, and 93.47 μg/mL for the FLP extract after 72 h of exposure. Significant cytotoxicity against breast cancer and colorectal cancer is consistent with data in the literature, although largely for organic solvent extracts of *S. aethiopis* and other Salvia species [16,53,54,55,56,57,58] and data on the cytotoxicity of aqueous extracts of *S. aethiopis* against these types of cancer is generally missing. Aqueous extracts of *Salvia miltiorrhiza* have been shown to inhibit the lung metastasis in an in vivo breast cancer model by inhibiting recruitment of M2-like macrophages via the CCL2-STAT3 axis [59].

Dissecting the individual chemical components that might underlie the above anticancer activity our HPLC results show that the extract of wild flowers and especially of wild leaves is particularly rich in rosmarinic acid. Numerous studies have validated rosmarinic acid’s anticancer potential, demonstrating its ability to selectively trigger cell death in malignant cells, to inhibit tumor growth, and restrict cancer cell proliferation and migration [60,61]. Rosmarinic acid has been shown to not only exert direct anticancer effects, but to also counteract tumor resistance to chemotherapy and to reduce tissue damage caused by chemo- and radiotherapy, mainly due to its radical scavenging capability. Proficient is also research reporting rosmarinic acid anticancer effects specifically against colorectal and breast cancer including cytotoxicity against the MCF-7 and HT-29 cancer cell lines examined in the present investigation with many studies elaborating on the mechanisms of these effects as well [60,61,62,63]. As extracts of wild flowers are slightly more efficient against all three types of cancer compared to extracts of wild leaves a question arises as to which particular component might contribute to this slightly higher efficiency. It could well be a contribution by caffeic acid which, according to our results, is present in the highest amount precisely in the extracts of wild flowers. Caffeic acid is well known for its anticancer activity stemming mainly from its ability to reduce oxidative stress, modulate cancer-related signaling pathways, and induce cancer cell death [64,65]. Particularly caffeic acid and its derivatives have been shown to be active against colon and colorectal cancer by modulating the phosphatidylinositide 3-kinases (PI3-K)/Akt, AMP-activated protein kinase (AMPK) and the mammalian target of rapamycin (m-TOR) signaling pathways [66] by increasing ROS generation and inducing mitochondrial membrane potential fall [67] by inhibiting survivin, a mitotic regulator with an important role in abnormal proliferation of cancer cells [68], and by polarizing macrophages toward an M1 phenotype, leading to pro-inflammatory cytokine secretion and the induction of Bax/Bcl 2-mediated colorectal cancer cell apoptosis [69] to name just a few of the mechanisms of caffeic acid anticancer effects. Similarly, clinical studies and experimental research based on MDA-MB-231 as well as MCF-7 cell lines demonstrate various anticancer properties of caffeic acid against both estrogen-receptor positive and estrogen-receptor negative breast cancer, including triple negative breast cancer [70,71,72,73].

The active anticarcinogenic components of the extract of wild flowers might also be other water soluble phenolic acids, reported for Salvia sp., represented by salvianolic acids A, B and C and salvianolic acid K with a structure derived from caffeic-acid-type units, similar to the other salvianolic acids (A, B, C, etc.) [74,75,76]. Data in the literature demonstrates the cytotoxicity of Sal A against MCF-7 cells underlined by the induction of apoptosis in the cells as well as its ability to hamper the migration and invasion of breast cancer cells and inhibit metastasis [77,78]. A further beneficial effect of Sal A is its ability to promote chemotherapy sensitivity which has already been shown in breast cancer for chemotherapeutic drugs such as paclitaxel and doxorubicin [79,80,81]. Likewise, Sal B induces apoptosis in breast cancer cells [81,82,83] and has been shown to decrease the tumor volume in an Ehrlich solid breast cancer model accompanied by a decrease in oxidative stress, inflammation and angiogenesis [83]. Additionally, research shows that Sal B induces cell death and triggers autophagy in HCT116 and HT29 colon cancer cells [84]. Moreover, Sal K, detected recently in *S. aethiopis* [43] and rosmarinic acid, a combination found to predominate in extracts from *Thymus zygis* subsp. Zygis, an endemic Portuguese plant, exhibit significant antiproliferative effects against the human colon adenocarcinoma cell line Caco-2 [85].

The present study demonstrates that extracts derived from *Salvia aethiopis* possess notable antiproliferative activity against several human cancer cell lines, including HeLa, HT-29, and MCF-7 cells. Importantly, the substantially higher IC_50_ values observed in the noncancerous BALB/3T3 fibroblast cell line indicate reduced toxicity towards normal cells, suggesting a degree of selectivity towards malignant cells. The MTT assay revealed a clear time-dependent reduction in the cancer cell viability following treatment with the tested extracts. Notably, HT-29 colorectal carcinoma cells exhibited the highest sensitivity to the extracts, particularly to flower extracts, which justified their selection as a model system for subsequent mechanistic investigations, including fluorescence microscopy and flow cytometric analyses.

Fluorescence microscopy analysis following acridine orange/ethidium bromide and DAPI staining revealed characteristic morphological features of apoptotic cell death in treated HT-29 cells, including chromatin condensation, nuclear fragmentation, and membrane blebbing. These observations were corroborated by Annexin V/PI flow cytometric analysis, which demonstrated a significant increase in apoptotic cell populations following treatment. Together, these results confirm that apoptosis represents a major mode of cell death induced by *S. aethiopis* extracts, consistent with previous observations for Salvia-derived extracts [86,87,88]. For example, extracts from *Salvia fruticosa* and *Salvia officinalis*, as well as the major phenolic compound rosmarinic acid, have been shown to inhibit proliferation and induce apoptosis in human colon carcinoma cell lines, at least in part through modulation of MAPK/ERK signaling pathways [87,88]. Rosmarinic acid has been demonstrated to exert its anticancer effects partially through the induction of apoptosis, as shown in other studies. It was reported to stimulate the expression of apoptosis-related factors such as Bax, caspase-3, and caspase-8, to inhibit the expression of anti-apoptotic proteins like Bcl-2 and PARP and to influence PI3K/Akt and NF-kB signaling pathways to provoke apoptosis in different cancer cell types [89]. Stimulation of apoptosis in various cancer cell lines has been observed for caffeic acid and its derivatives as well [64,66,67,69]. In line with previous studies analyzing the types of cell death induced by Salvia extracts [43,54], the present study also demonstrated that, in addition to apoptosis, treatment with *S. aethiopis* extracts resulted in an increase in necrotic cell populations. Similar observations have been reported for *Rosmarinus officinalis* (syn. *Salvia rosmarinus*), where necrosis was proposed as a major mechanism of colon cancer cell death. Mechanistically, this effect has been associated with ROS-mediated disruption of cellular homeostasis, leading to endoplasmic reticulum stress and activation of the unfolded protein response (UPR), along with modulation of PI3K/Akt and Nrf2 signaling pathways [54]. These alterations may initially trigger responses such as apoptosis and autophagy; however, when cellular stress exceeds the compensatory capacity of these mechanisms, the balance may shift towards necrotic cell death. This transition may be linked to mitochondrial dysfunction, a key determinant of cellular fate under stress.

Consistent with this, JC-1 staining revealed disruption of mitochondrial membrane potential in the present study, supporting the central role of mitochondria in these processes. Mitochondrial depolarization represents an early event in the intrinsic apoptotic pathway and is associated with mitochondrial outer membrane permeabilization and the release of cytochrome c, leading to the activation of caspase-dependent signaling cascades [90]. The observed decrease in the proportion of cells with polarized mitochondria following treatment is therefore consistent with activation of mitochondria-mediated apoptosis. Similar mechanisms have been reported for other species of the genus Salvia, where plant extracts were shown to induce apoptosis through mitochondrial and caspase-mediated pathways in cancer cells [86,91,92]. Rosmarinic acid has also been shown to reduce mitochondrial membrane potential and promote mitochondrial fission with concomitant activation of intrinsic apoptotic pathways [93]. In a similar way the anticancer effect of caffeic acid and its derivatives has been associated with triggering mitochondrial dysfunction in cancer cells [94,95]. In addition to the induction of cell death through both apoptotic and necrotic pathways, the extracts significantly affected cell cycle progression. Flower extracts from wild-growing plants caused a marked decrease in the proportion of S-phase cells, accompanied by a moderate increase in the G2/M population, suggesting checkpoint dysregulation and aberrant mitotic progression. In contrast, extracts from in vitro-propagated plants led to a significant reduction in the G1 population and a pronounced increase in the >4 N fraction, indicative of mitotic defects and potential mitotic failure. Together, these findings indicate that multiple levels of cell cycle dysregulation contribute to the overall antiproliferative effect.

Further insight into the cellular response to *S. aethiopis* extracts was provided by fluorescence microscopy analysis using MDC and DR staining which provided evidence for the involvement of autophagy-related processes in the cellular response to *S. aethiopis* extracts. Treatment of HT-29 cells with the extracts resulted in a pronounced increase in DR fluorescence intensity, indicating a substantial expansion or activation of acidic vesicular compartments. In contrast, MDC staining showed only a moderate increase, suggesting a relatively limited accumulation of autophagic vacuoles. Notably, an increased overlap between MDC and DR signals was observed in treated cells, indicating partial colocalization of autophagic vesicles with acidic lysosomal compartments. The marked elevation in DR fluorescence, together with the modest increase in MDC staining, suggests that the extracts may preferentially enhance late-stage autophagy-related processes, such as autophagosome–lysosome fusion and autolysosome formation, rather than strongly inducing early autophagosome formation. These findings suggest that *S. aethiopis* extracts may modulate autophagy in HT-29 cells. The concurrent induction of apoptosis and modulation of autophagy-related vesicular dynamics observed in this study is consistent with previous reports describing coordinated activation of these processes in response to Salvia extracts [96,97]. In particular, salvianolic acid B, as mentioned previously has been shown to induce autophagy and promote cell death in colorectal cancer cells, including HT-29, through modulation of the AKT/mTOR signaling pathway [84].

Apigenin, another compound detected in the extracts, has also been widely reported to exert anticancer effects in colorectal cancer through the modulation of key signaling pathways such as PI3K/Akt/mTOR, MAPK/ERK, Wnt/β-catenin, and STAT3. It has been shown to induce G2/M cell cycle arrest, promote apoptosis, and regulate autophagy in colorectal cancer cells [98,99], which is consistent with the cell cycle alterations and induction of cell death observed in the present study.

The results of the anticancer activity study indicate that *S. aethiopis* extracts exert pronounced cytotoxic effects in colorectal carcinoma cells through coordinated modulation of multiple cellular processes, including mitochondrial dysfunction, apoptosis induction, cell cycle disruption, and modulation of autophagy-related pathways. The higher activity observed for extracts derived from wild-growing plants is likely associated with their richer phytochemical composition, particularly the higher content of rosmarinic acid and related phenolic compounds. These findings highlight *S. aethiopis* as a promising source of bioactive molecules with potential applications in colorectal cancer therapy and warrant further investigation into their molecular targets and in vivo efficacy.

## 4. Materials and Methods

### 4.1. In Vitro Culture

#### 4.1.1. Initial Plant Material

Ripe seeds of *Salvia aethiopis* L. were collected from natural populations in the Slavyanka Mountain (Bulgaria) at the end of September 2022.

#### 4.1.2. Sterilization of Plant Material

Seeds were thoroughly washed with detergent under running tap water and surface-sterilized using three different sterilization protocols: (1) 70% ethanol for 2 min followed by 0.1% HgCl_2_ for 10 min; (2) 70% ethanol for 2 min followed by treatment with 100% bleach solution (containing 4.85% available chlorine); and (3) 70% ethanol for 2 min followed by treatment with 20% bleach solution (containing 4.85% available chlorine). After disinfection, the seeds were rinsed with sterile distilled water for 5, 10, or 15 min to remove residual sterilizing agents. The sterilized seeds were subsequently germinated on full-strength Murashige and Skoog (MS) [21] medium solidified with 0.7% agar and supplemented with 2% sucrose.

#### 4.1.3. Media Composition for In Vitro Micropropagation

The single-node explants (1.0–1.5 cm in length) were inoculated on MS medium supplemented with various concentrations of 6-benzylaminopurine (BAP) (0.5 or 1 mg/L) and kinetin (K) (1 mg/L) applied alone. The other tested MS media containing BAP, KIN or zeatin (Z) (1 mg/L) in combination with Indole-3-acetic acid (IAA) (0.1 mg/L) or Indole-3-butyric acid (IBA) (0.1 mg/L). The percentage of shoot induction [%], the average number of shoots per explant and the mean shoot height were measured after 4 weeks of culture. The composition of nutrient medium was given in Table 1.

#### 4.1.4. In Vitro Rooting and Acclimatization of Obtained Plants

To induce roots regenerated shoots were placed on half strength MS nutrient medium supplemented with indole-3-butyric acid (0.1; 0.2; 0.25 mg/L) or in combination with 0.2 mg/L IAA and 2 mg/L IBA (Table 2).

The rooted shoots were moved to plastic pots (8 cm in diameter) and filled with soil, peat, perlite, and sand (2:1:1:1) after being gently washed with tap water. For two weeks, the pots were covered (shielded) with clear plastic boxes to ensure that the plants had a high relative humidity. Additional culture conditions were 50 μM/m^2^/s light, 16/8 photoperiod, and 25 °C. Plants were moved to the greenhouse five weeks after adaption and after the survival rate was recorded.

#### 4.1.5. Conditions for In Vitro Cultures

In vitro plant materials were cultivated in a growth room with artificial illumination (lamps type fluorescent—FL^−40^W^−1^, Svetlina Ltd., Stara Zagora, Bulgaria) under a 16 h photoperiod at 18–21 °C, with a photon flux density of 40 μM/m^2^/s for shoot formation and 20 μM/m^2^/s for root initiation and maintenance.

### 4.2. Plant Material Extraction and Analysis

#### 4.2.1. Plant Material

Aerial parts from both wild plants (collected from Slavyanka Mountain) and in vitro-cultivated specimens grown in an experimental field were harvested. The plant material was collected at the end of the vegetation period (June–July, year 2024) and air-dried in the shade at ambient temperature. Following drying, leaves, flowers, and stems were separated, placed in paper bags, and stored at room temperature.

#### 4.2.2. Preparation of Freeze-Dried Extracts

Dried plant materials were milled to fine powder in a laboratory mill. Five grams of the powder was extracted for 15 min in 200 mL of water (90 °C). The resulting slurry was centrifuged at 6000× *g* (MPW-260R, MPW Med. Instruments, Warszawa, Poland), and the supernatants were collected and freeze dried for 96 h in an Alpha 1–4 LD plus laboratory freeze drier (Martin Christ Gefriertrocknungsanlagen GmbH, Osterode am Harz, Germany). The freeze-dried extracts were denoted as FCP—flowers from in vitro-cultivated plants, LCP—leaves from in vitro-cultivated plants, SCP—stems from in vitro-cultivated plants, FWP—flowers from wild plants, LWP—leaves from wild plants, and SWP—stems from wild plants, and were used for total polyphenol and flavonoid content, antioxidant activity determination and assessment of antitumor activity.

#### 4.2.3. Total Polyphenols and Flavonoid Content

Total polyphenol content was determined according to Singleton and Rossi [100]. Gallic acid was used as the calibration standard, and the results are expressed as mg gallic acid equivalents (GAE) per 100 g dry weight (DW) ± SD.

The total flavonoid content was assessed with aluminum chloride (AlCl_3_) reagent, according to the method of Chang et al. [101]. A calibration curve was generated with rutin, and the results are expressed as mg quercetin equivalents (QE) per 100 g DW ± SD.

#### 4.2.4. Antioxidant Activity

Oxygen radical absorbance capacity (ORAC) activity was measured on a microplate reader (FLUOstar OPTIMA; BMG Labtech, Ortenberg, Germany) according to the method of Ou et al. [102], with some modifications described by Denev et al. [103]. The results are expressed in micromole trolox equivalents (μmol TE) per gram DW ± SD.

The hydroxyl radical averting capacity (HORAC) activity of the freeze-dried extract was determined according to Ou et al. [104]. The results are expressed in micromole gallic acid equivalents (μmol GAE) per gram DW ± SD.

#### 4.2.5. HPLC Determination of Phenolic Compounds

HPLC analyses were performed according to Atanasova et al. [105] on a UHPLC system Nexera-i LC2040C Plus (Shimadzu Corporation, Kyoto, Japan) with a UV-VIS detector and a binary pump. The column was a Shimadzu Shim-pack GIST-C18, 5 µm, 4.6 × 250 mm (Shimadzu Corporation, Kyoto, Japan), thermostated at 30 °C. The flow rate was 0.3 mL/min and the injection volume was 5 μL. The derivatives were detected at λ = 280 nm. The mobile phase consisted of A: 0.5% acetic acid and B: 100% acetonitrile. The gradient condition started with 14% (B), between 6 and 30 min, linearly increased to 25% (B), and then to 50% (B) at 40 min. The identification of compounds was confirmed by a comparison of retention times utilizing standard solutions and standard calibration curves of different phenolics (gallic acid, neochlorogenic acid, 3,4-di-hydroxy benzoic acid, chlorogenic acid, catechin, vanillic acid, caffeic acid, epicatechin, p-coumaric acid, ferulic acid, rutin, ellagic acid, quercetin-3-β-glucoside, naringin, rosmarinic acid, myricetin, cinnamic acid, quercetin, luteolin, naringenin, apigenin and kaempferol). The results for individual phenolic compounds were expressed in mg per 100 g DW ± SD.

### 4.3. Antitumor Activity

#### 4.3.1. Cell Culture Conditions

Human cancer cell lines, HeLa (cervical carcinoma), HT-29 (colorectal adenocarcinoma), and MCF-7 (breast adenocarcinoma), together with the non-tumorigenic murine fibroblast cell line BALB/3T3, were obtained from the American Type Culture Collection (ATCC, Manassas, VA, USA). Cells were maintained in Dulbecco’s Modified Eagle’s Medium (DMEM, Gibco, Grand Island, NY, USA) supplemented with 10% fetal bovine serum (FBS), 2 mM L-glutamine, 100 U/mL penicillin, and 100 μg/mL streptomycin. Cultures were incubated at 37 °C in a humidified atmosphere containing 5% CO_2_. Cells were subcultured using trypsin–EDTA and experiments were conducted with cells in the exponential growth phase.

#### 4.3.2. Evaluation of Cell Viability

The antiproliferative effects of the plant extracts were determined using the MTT colorimetric assay. Briefly, cells were harvested, counted with a hemocytometer, and seeded in 96-well plates at a density of 1 × 10^4^ cells per well in 100 μL complete medium. Following overnight attachment, cells were exposed to increasing concentrations of the *S. aethiopis* plant extracts (125–1000 μg/mL) for 24 or 48 h. Untreated cells grown under identical conditions served as negative controls, while BALB/3T3 fibroblasts were used to assess selectivity toward malignant cells. Each concentration was tested in five independent replicates. After treatment, cells were washed with phosphate-buffered saline (PBS) and incubated with MTT solution (5 mg/mL) for 3 h at 37 °C. Formazan crystals were subsequently dissolved in a DMSO/ethanol mixture (1:1, *v*/*v*), and absorbance was measured at 570 nm using a microplate reader (TECAN, Sunrise™, Grödig, Austria). Cell viability was expressed as a percentage relative to untreated controls. IC_50_ values were calculated by nonlinear regression analysis.

#### 4.3.3. Fluorescence Microscopy Analysis

To investigate extract-induced morphological and cytotoxic changes, HT-29 colorectal carcinoma cells were grown on sterile glass coverslips placed in 24-well plates and treated for 24 h with extract concentrations corresponding approximately to the IC_50_ values determined by the MTT assay. Following treatment, cells were subjected to different fluorescent staining protocols and examined using a Leica DM 5000B fluorescence microscope.

##### Acridine Orange/Ethidium Bromide Staining

Cell membrane integrity and the viability of extract-treated cancer cells were assessed using acridine orange (AO) and ethidium bromide (EtBr) double staining. Treated and control cells were incubated with AO (5 μg/mL) and EtBr (5 μg/mL) in PBS and immediately visualized using a fluorescence microscope (Leica DM 5000B, Wetzlar, Germany) to distinguish viable, apoptotic, and necrotic cells based on fluorescence emission patterns.

##### Nuclear Morphology Assessment by DAPI

Extract-induced nuclear alterations associated with apoptosis were evaluated using 4′,6-diamidino-2-phenylindole (DAPI) staining. Cells were fixed with ice-cold methanol, incubated with DAPI solution (1 μg/mL) in the dark for 15 min, washed, and mounted for fluorescence microscopy observation. Fluorescence microscopy images were taken using a Leica DM 5000B fluorescence microscope (Wetzlar, Germany).

#### 4.3.4. Annexin V/Propidium Iodide Double Staining Fowl Cytometry

For the analysis of the proapoptotic potential of the flower extracts of wild-growing and in vitro-propagated *S. aethiopis* plants, an Annexin V-FITC/PI Apoptosis Detection Kit (Santa Cruz Biotechnology, Dallas, TX, USA) was used. HT-29 cells at a density of 1 × 10^5^ cells/well were seeded in 6-well plate and treated with the tested extracts for 24 h. Non-treated cells were used as controls. Then the cells were trypsinized with Trypsin-EDTA, centrifuged for 10 min at 1000 rpm and washed twice with PBS. After that, they were suspended in binding buffer (0.01 M Hepes/NaOH, pH 7.4 supplied with 0.14 M NaCl and 2.5 mM CaCl_2_). Annexin V-FITC (5 µL) and PI (5 µL) were added to 100 µL of each cell suspension. After incubation for 15 min at RT, 10,000 cells were analyzed from each sample with a Becton Dickinson flow cytometer (BD Bio-sciences, San Jose, CA, USA) and Diva 6.1.1. software.

#### 4.3.5. Cell Cycle Distribution Analysis

Cell cycle progression was examined by flow cytometry following extract treatment. Cells were seeded in culture plates, allowed to attach, and treated with selected extract concentrations for 24 h. Cells were then harvested, washed with cold PBS, and fixed in 70% ethanol added dropwise under continuous vortexing. Fixed cells were stored at −20 °C for at least 12 h. Before analysis, samples were washed, incubated with RNase A (20 μg/mL) for 30 min, and stained with propidium iodide (20 μg/mL). DNA content was analyzed using a Becton Dickinson flow cytometer, with at least 10,000 events recorded per sample. Cell cycle phase distribution (G_0_/G_1_, S, and G_2_/M) was quantified using FlowJo™ software (v10.8). Results are presented as mean ± SEM from three independent experiments.

#### 4.3.6. Assessment of Mitochondrial Membrane Potential

Changes in mitochondrial membrane potential (ΔΨm) were evaluated using the JC-1 fluorescent probe. After 24 h of extract treatment, cells were incubated with JC-1 dye (5 μg/mL) for 20 min at 37 °C in the dark. Fluorescence images were captured by fluorescence microscopy, and mitochondrial depolarization was assessed by calculating the ratio of red (JC-1 aggregates) to green (JC-1 monomers) fluorescence intensity. For each treatment group, 50 cells were analyzed using ImageJ software version 1.50i, and the relative red and green fluorescence intensities were determined by dividing the integrated density by the area. The results are presented as the ratio of relative red to green fluorescence, expressed as the mean value from three independent experiments.

#### 4.3.7. Assessment of Autophagy Induction

Autophagy induction in HT-29 colorectal carcinoma cells following treatment with flower extracts from wild-growing and in vitro-propagated *S. aethiopis* plants was evaluated by fluorescence microscopy using 50 nM monodansylcadaverine (MDC) and 10 µM LysoTracker Deep Red (DR) staining. MDC preferentially accumulates in autophagic vacuoles due to its affinity for autophagic membranes, while DR labels acidic compartments such as lysosomes and autolysosomes. The simultaneous visualization of MDC-positive vesicles and DR fluorescence allows detection of autophagic structures and lysosomal activity associated with autophagy. The stained control and treated cells were examined and captured using a Leica DM fluorescence microscope (5000B, Wetzlar, Germany). The intensity of green and red fluorescence was quantified using ImageJ software, and the results were expressed as fold change relative to the untreated control cells.

### 4.4. Statistical Analysis

All data are expressed as mean ± standard deviation (SD). Statistical comparisons were performed using one-way analysis of variance (ANOVA) followed by Bonferroni’s post hoc test in GraphPad Prism 8.0 software. Differences were considered statistically significant at *p* < 0.05.

## 5. Conclusions

In the present study, an efficient protocol for the in vitro propagation of *Salvia aethiopis* was developed. Optimal shoot multiplication was achieved on MS medium supplemented with kinetin and indole-3-acetic acid (IAA), while the highest rooting efficiency was obtained on half-strength MS medium containing indole-3-butyric acid (IBA). The phytochemical composition and biological activity of extracts obtained from leaves, flowers, and stems of both in vitro-cultivated and wild plants were evaluated and compared. HPLC analysis confirmed the presence of key bioactive compounds, including rosmarinic acid, caffeic acid, and apigenin, while ORAC and HORAC assays demonstrated notable antioxidant capacity. The extracts exhibited significant cytotoxicity against cervical, mammary, and colorectal carcinoma cell lines, while noncancerous cells were considerably less affected. The flower extract of wild plants showed the highest anticancer activity, with IC_50_ values below 100 μg/mL for all tested cancer cell lines, along with the highest polyphenol and flavonoid content and the strongest antioxidant activity among all extracts. Mechanistic studies demonstrated that the anticancer effects of *S. aethiopis* extracts were mediated by inhibition of proliferation, mitochondrial dysfunction, cell cycle disruption, autophagy-related responses, and induction of both apoptosis and necrosis. These findings highlight *S. aethiopis* as a promising candidate for anticancer therapy, warranting further validation through more detailed mechanistic and metabolomics studies.

## Figures and Tables

**Table 1:** Effect of plant growth regulators on micropropagation of *S. aethiopis*.

Plant Growth Regulators [mg/L]	Shoot Formation [%]	Mean Number of Shoot Per Explant	Shoot Height [cm]
BAP_0.5_	20	1.25 ± 0.25 ^a^	2.80 ± 0.08 ^c^
BAP_1_	70	1.71 ± 0.16 ^b^	2.79 ± 0.12 ^c^
BAP_1_ + IBA_0.1_	30	1.33 ± 0.21 ^a^	2.20 ± 0.22 ^a^
BAP_1_ + IAA_0.1_	90	3.61 ± 0.18 ^e^	3.70 ± 0.18 ^e^
K_1_	70	2.64 ± 0.22 ^c^	2.33 ± 0.15 ^ab^
K_1_ + IBA_0.1_	60	3.08 ± 0.19 ^d^	3.10 ± 0.15 ^d^
K_1_ + IAA_0.1_	100	4.00 ± 0.17 ^f^	3.60 ± 0.11 ^e^
Z_1_ + IBA_0.1_	50	2.60 ± 0.22 ^c^	2.51 ± 0.19 ^b^
Z_1_ + IAA_0.1_	80	3.33 ± 0.11 ^de^	2.33 ± 0.08 ^ab^
LSD	-	0.33	0.25

Legend: Data are presented as mean values of 40 buds with the mean being shown as mean ± standard error (SE). The letters “a”, “b”, “c”, “d”, “e”, and “f” next to the values in the table refer to the statistical groupings as a result of Fisher’s LSD test (*p* ≤ 0.05) applied after one-way ANOVA. When the two values share the same letter, no significant difference is demonstrated; a significant difference is denoted only by different letters next to the values.

**Table 2:** In vitro rooting of *S. aethiopis*.

Nutrient Medium [mg/L]	Rooting [%]	Mean Number of Roots Per Explants	Root Length [cm]
IBA_0.1_	30	2.30 ± 0.21 ^a^	2.30 ± 0.22 ^b^
IBA_1_	20	2.25 ± 0.25 ^a^	1.60 ± 0.08 ^a^
IBA_0.25_	50	4.50 ± 0.16 ^b^	3.60 ± 0.17 ^d^
IAA_0.2_ + IBA_2_	40	2.37 ± 0.18 ^a^	3.00 ± 0.11 ^c^
LSD	-	0.38	0.29

Legend: Data are presented as mean values of 40 buds with the mean being shown as mean ± standard error (SE). The letters “a”, “b”, “c” and “d” next to the values in the table refer to the statistical groupings as a result of Fisher’s LSD test (*p* ≤ 0.05) applied after one-way ANOVA. When the two values share the same letter, no significant difference is demonstrated; a significant difference is denoted only by different letters next to the values.

**Table 3:** Total polyphenol and flavonoid content and antioxidant activity of extracts from different anatomical parts of in vitro-obtained field-grown and wild *S. aethiopis* plants.

Sample	Total Polyphenols [mg GAE/100 g DW]	Total Flavonoids [mg RE/100 g DW]	ORAC [µmol TE/g DW]	HORAC [µmol GAE/g DW]
FCP	10,300 ± 256 ^a^	1456 ± 85 ^a^	3443 ± 246 ^a^	801 ± 64 ^a^
FWP	14,681 ± 211 ^a^	2317 ± 77 ^b^	4563 ± 280 ^b^	1233 ± 63 ^a^
LCP	11,127 ± 148 ^a^	1723 ± 101 ^c^	3459 ± 157 ^bc^	900 ± 56 ^b^
LWP	9303 ± 165 ^a^	1704 ± 90 ^d^	3235 ± 191 ^c^	724 ± 42 ^c^
SCP	9356 ± 190 ^ab^	178 ± 17 ^d^	3001 ± 111 ^d^	699 ± 57 ^c^
SWP	11,756 ± 255 ^b^	909 ± 65 ^c^	3930 ± 201 ^c^	935 ± 55 ^d^
LSD	415	79.7	211.1	58.1

Legend: FCP (flowers of in vitro-cultivated plants); FWP (flowers of wild plants); LCP (leaves of in vitro-cultivated plants); LWP (leaves of wild plants); SCP (stems of in vitro-cultivated plants); SWP (stems of wild plants). The letters “a”, “b”, “c” and “d” next to the values in the table refer to the statistical groupings as a result of Fisher’s LSD test (*p* ≤ 0.05) applied after one-way ANOVA. When the two values share the same letter, no significant difference is demonstrated; a significant difference is denoted only by different letters next to the values.

**Table 4:** Content of main phenolic compounds (mg/100 g DW) of extracts from different anatomical parts of in vitro-obtained field-grown and wild *S. aethiopis* plants.

Sample	Caffeic Acid [Mean ± SD]	Rosmarinic Acid [Mean ± SD]	Apigenin [Mean ± SD]
FCP	56.2 ± 7.6 ^a^	1249.0 ± 101.9 ^a^	17.1 ± 0.5 ^a^
FWP	93.2 ± 6.8 ^a^	3336.3 ± 217.4 ^a^	51.1 ± 2.6 ^ab^
LCP	34.1 ± 3.2 ^a^	1925.4 ± 163.0 ^b^	75.4 ± 7.0 ^b^
LWP	67.1 ± 9.7 ^b^	5157.1 ± 255.9 ^c^	268.8 ± 18.5 ^c^
SCP	36.9 ± 0.8 ^c^	1084.6 ± 106.2 ^d^	13.1 ± 0.8 ^d^
SWP	40.0 ± 1.3 ^d^	2450.7 ± 154.0 ^c^	24.0 ± 0.7 ^c^
LSD	6.07	180.5	8.38

Legend: FCP (flowers of in vitro-cultivated plants); FWP (flowers of wild plants); LCP (leaves of in vitro-cultivated plants); LWP (leaves of wild plants); SCP (stems of in vitro-cultivated plants); SWP (stems of wild plants). The letters “a”, “b”, “c” and “d” next to the values in the table refer to the statistical groupings as a result of Fisher’s LSD test (*p* ≤ 0.05) applied after one-way ANOVA. When the two values share the same letter, no significant difference is demonstrated; a significant difference is denoted only by different letters next to the value.

**Table 5:** Inhibitory concentrations (IC_50_, μg/mL) of plant extracts obtained from flowers, leaves, and stems of in vitro-propagated and wild-growing *S. aethiopis* plants, calculated based on MTT cell viability data.

Cell Line	FWP	FCP	LWP	LCP	SWP	SCP
HeLa 24 h	98.7	228.2	128.7	285.9	279.1	605.9
HeLa 72 h	73.8	167.5	93.47	263.5	213.2	565.2
HT-29 24 h	175.2	391.5	157.4	453.9	433.5	>1000
HT-29 72 h	50.4	144.7	67.01	187.9	177.0	451.5
MCF-7 24 h	273.1	435.7	208.2	640.6	682.1	>1000
MCF-7 72 h	65.7	179.9	74.9	211.3	181.9	435.4
BALB/3T3 24 h	493.1	757.1	>1000	>1000	>1000	>1000
BALB/3T3 72 h	230.9	455.1	240.9	592.2	479.9	917.7

Legend: FCP (flowers of in vitro-cultivated plants); FWP (flowers of wild plants); LCP (leaves of in vitro-cultivated plants); LWP (leaves of wild plants); SCP (stems of in vitro-cultivated plants); SWP (stems of wild plants).

## Data Availability

All data are comprised in the manuscript.
